# Unhealthy visceral fat is associated with improved efficacy of immunotherapy in endometrial cancer

**DOI:** 10.1172/JCI183675

**Published:** 2024-09-03

**Authors:** Matthew L. Steinhauser

**Affiliations:** 1 Center for Human Integrative Physiology, Aging Institute, University of Pittsburgh School of Medicine, Pittsburgh, Pennsylvania, USA.; 2 Division of Cardiovascular Medicine, Department of Medicine, University of Pittsburgh Medical Center and School of Medicine, Pittsburgh, Pennsylvania, USA.

## Abstract

Obesity is a known driver of endometrial cancer. In this issue of the *JCI,* Gómez-Banoy and colleagues investigated a cohort of patients with advanced endometrial cancer treated with immune checkpoint inhibitors targeting the interaction between programmed cell death receptor-1 (PD-1) and its ligand (PD-L1). Notably, a BMI in the overweight or obese range was paradoxically associated with improved progression-free and overall survival. A second paradox emerged from CT analyses of visceral adipose tissue, viewed as an unhealthy fat depot in most other contexts, the quantity of which was also associated with improved treatment outcomes. Though visceral adiposity may have value as a biomarker to inform personalized treatment strategies, of even greater impact would be if a therapeutic strategy emerges from the future identification of adipose-derived mediators of this putative anticancer immune-priming effect.

## The importance of context

Obesity is a driver of diverse pathologies, including cardiometabolic diseases and cancer, with endometrial cancer being among the most strongly associated (1, 2). However, the excess adiposity and underlying mechanisms that define overweight and obese states are not positive predictors of pathological outcomes in all contexts. Indeed, the very predisposition to obesity in humans has been advanced as a modern reflection of our changing evolutionary environment. The “thrifty gene hypothesis,” as proposed by James Neel in the 1960s, posits that metabolic and energetic efficiencies that promote obesity were selected for in the context of cyclic famine that characterized our evolutionary past (3). Obesity is a risk factor for heart failure, yet in some studies, obesity is associated with attenuated risk of death in patients with established disease (4). While overweight and obesity are generally associated with excess mortality, some epidemiological studies in older adults suggest a U-shaped curve, with optimal longevity at a BMI in the overweight range (5). While many so-called “obesity paradoxes” remain controversial due to inconsistency across studies coupled with the correlative nature of the underlying evidence, these collective examples point to the importance of context even with a condition as tightly linked to disease as obesity.

## Higher BMI and endometrial cancer

The advent of immune checkpoint inhibitors as a therapeutic strategy to augment the endogenous anticancer immune response has revealed another obesity paradox, as a higher BMI predicts treatment efficacy in several malignancies (6–8). In this issue of the *JCI,* Gómez-Banoy and colleagues now extend this paradox to endometrial cancer treated with checkpoint inhibitors targeting the interaction between programmed cell death receptor-1 (PD-1) and its ligand (PD-L1) (9). PD-1 is expressed on T cells. When bound to PD-L1, a protein often overexpressed by tumor cells, PD-1 suppresses T cell activation, thereby promoting immune evasion by PD-L1–expressing cells. Disruption of PD-1/PD-L1 interaction by one of several currently available antibody-based therapeutics is the front-line therapy for advanced endometrial cancer. Gómez-Banoy and investigators retrospectively identified 768 patients with endometrial cancer treated with immune checkpoint inhibitors between 2015 and 2022, of whom 524 met criteria of recurrent, advanced, or metastatic endometrial cancer and hence were included in their formal analyses. Most patients in the cohort were treated with anti–PD-1 therapy (85%), with 15% receiving anti–PD-L1 therapy. Recognizing that endometrial cancer is a heterogeneous disease, the authors selected a cohort that generated a sufficiently broad distribution of clinical characteristics to facilitate multivariate analyses. They confirmed that the improved progression-free and overall survival of patients with an elevated BMI in the overweight or obese range was not driven by obvious potential confounders, such as cancer stage, prior therapeutic exposures, or histological or molecular subtypes. Indeed, the relationship between BMI categories and treatment efficacy was strongest in patients with the copy number–high/TP53abnormal (CN-H*/TP53abn*) molecular subtype, which has been associated with immune cells expressing PD-1/PD-L1 in tumors. These findings provide conceptual support for obesity as having an on-target effect on treatment responsiveness. Despite limitations inherent to the retrospective study design, the reasonable size and diversity of the cohort coupled with appropriate multivariate adjustments for likely confounders provides robust evidence for an obesity paradox in patients with endometrial cancer undergoing treatment with immune checkpoint inhibitors (9).

The Gómez-Banoy study also breaks important ground by moving beyond a simple BMI-centric view of obesity to a consideration of adiposity traits (9). This nuanced viewpoint is important because BMI does not account for differences in lean mass, nor does it reflect the recognition of depot-dependent differences in fat biology that has emerged from mechanistic studies in rodents and humans. The predilection for visceral adipose tissue (VAT), for example, is heritable and its underlying genetic architecture appears distinct from the genetics of obesity itself (10). VAT exhibits greater potential for lipolytic release of fatty acids and poor capacity for metabolically active beige/brown fat phenotypes relative to subcutaneous adipose tissue (SAT) (11, 12). VAT also displays a greater capacity for new fat cell formation and an obesity-related cycle of fat cell death and regeneration, which is associated with increased macrophage influx to resolve lipid-rich dead adipocytes (13–15). Macrophages contained in VAT with obesity appear particularly prone to a proinflammatory cell state, whereas counterbalancing regulatory lymphocyte populations (e.g., T-regulatory cells) are diminished in number and/or function in obese VAT (16, 17). Increased VAT is further associated with a systemic imbalance in potentially toxic proinflammatory cytokines relative to protective antiinflammatory cytokines and adipokines such as adiponectin (11). It is thought that such VAT-specific pathobiology accounts for why VAT volume, as can be measured by clinical imaging, predicts cardiometabolic and cancer risk (18). Most patients (*n* = 500, 95%) in the Gómez-Banoy cohort (9) had clinical CT scans of the abdomen available for 2D measurement of SAT and VAT quantity at the L3/L4 spine level, which generally correlates with volumetric measures and clinical outcomes (19). This analysis identified a second interesting paradox in that the quantity of putative unhealthy VAT, and not SAT, predicted positive clinical responses to immunotherapy.

## An informative paradox

**Figure 1:**
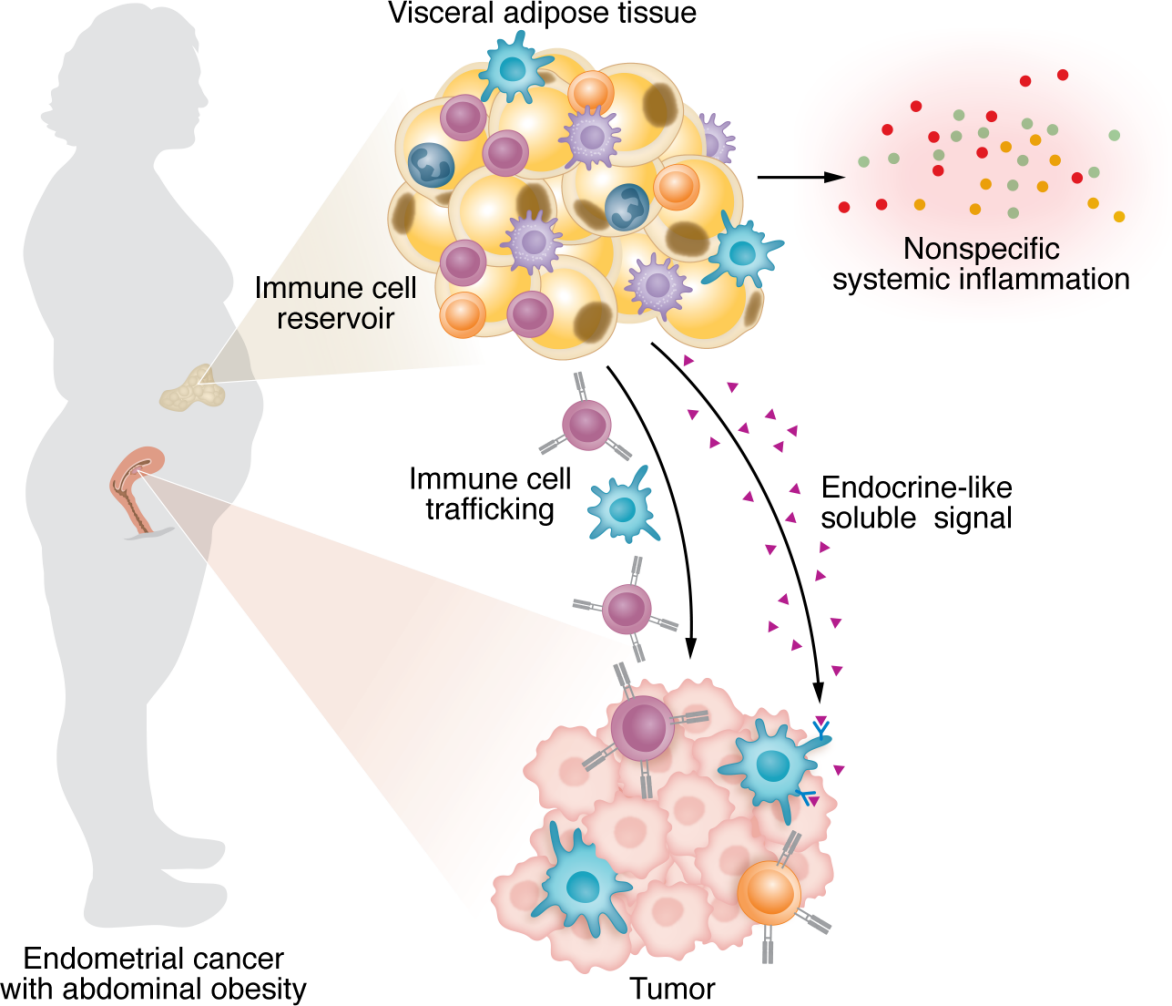
VAT may prime antitumor immunity in women with endometrial cancer and obesity. Three potential mechanisms explain the paradox underlying the relationship between putative unhealthy VAT and positive clinical responses to immunotherapy in endometrial cancer with abdominal obesity. VAT is a known contributor to a generalized state of chronic sterile metabolic inflammation, in part through systemic release of various inflammatory signals. There may also be a more specific signal emanating from VAT that acts distantly on specific immune cell types in the tumor. Finally, VAT is replete with immune cells and therefore is a theoretical reservoir supporting immune cell trafficking to the tumor site.

Given the immunological profile of VAT, an interesting possibility emerges that some property of VAT might prime antitumor immune responses (Figure 1). There are several possibilities for how such a mechanism might work. (a) As discussed, VAT is associated with increased systemic sterile inflammation, and antitumor responses could gain a nonspecific advantage. Indeed, the Gómez-Banoy study (9) found that obesity predicted immune-related adverse events, consistent with a more generalized immune-priming effect as shown previously (20). However, the association with extratumor immune activity did not hold for VAT. (b) Adipose tissue is one of the largest tissues in the body and, given its diverse immune cell constituents, it is possible that VAT is a reservoir for antitumor effector cells. Though not shown in the context of obesity, emerging evidence suggests that lymphocytes can efflux from peripheral tissues back into circulation and therefore theoretically could in turn home to tumor tissue (21). (c) Beyond canonical inflammatory mediators, adipose tissue and adipocytes release additional bioactive soluble products, including hormones, peptides, lipids, and extracellular vesicles containing diverse payloads with signaling potential. Indeed, adipocyte-derived leptin was previously shown to be a potential determinant of T cell functions relevant to cancer in obesity (7); however, any one of these soluble mediators could in theory mediate direct crosstalk between adipose tissue and tumor-associated immune cells.

In summary, Gómez-Banoy et al. demonstrate that in the narrow context of immune check point inhibitor treatment of endometrial cancer, a higher BMI paradoxically predicts treatment efficacy and positive clinical outcomes (9). The authors are appropriately cautious about assigning causality, given the retrospective study design and the lack of blood and tissue samples that would enable a more granular dissection of potential mechanisms. However, the diversity of cancer types for which the therapeutic efficacy of immunotherapies is improved with obesity increases the likelihood that such a mechanism exists. It is the additional paradoxical discovery of a potential mediator in VAT — an adipose depot viewed as unhealthy in most other contexts — that may ultimately be the most impactful aspect of this study, as the future identification of a fat-derived immune-priming signal could serve equally as a biomarker to guide personalized treatments and/or a novel therapeutic path to synergize with anticancer immunotherapies more broadly.

## Version 1 09/03/2024

Electronic publication
